# Next-generation sequencing-based clinical diagnosis of choroideremia and comprehensive mutational and clinical analyses

**DOI:** 10.1186/s12886-020-01478-x

**Published:** 2020-06-01

**Authors:** Feng-Juan Gao, Guo-Hong Tian, Fang-Yuan Hu, Dan-Dan Wang, Jian-Kang Li, Qing Chang, Fang Chen, Ge-Zhi Xu, Wei Liu, Ji-Hong Wu

**Affiliations:** 1 Eye Institute, Eye and ENT Hospital, Fudan University, Shanghai, 200032 China; 2 Shanghai Key Laboratory of Visual Impairment and Restoration, Science and Technology Commission of Shanghai Municipality, Shanghai, China; 3 State Key Laboratory of Medical Neurobiology, Institutes of Brain Science and Collaborative Innovation Center for Brain Science, Shanghai Medical College, Fudan University, Shanghai, China; 4 NHC Key Laboratory of Myopia, Fudan University, Shanghai, China; 5 BGI-Shenzhen, Shenzhen, Guangdong China; 6 BGI-Changyuan, Xinxiang, Henan China; 7 BGI Education Center, University of Chinese Academy of Sciences, Shenzhen, China

**Keywords:** Choroideremia, *CHM*, Gene mutations, Molecular diagnosis, Optical coherence tomography

## Abstract

**Background:**

To report the clinical and genetic findings from seven Chinese patients with choroideremia.

**Methods:**

Five hundred seventy-eight patients with a clinically suspected diagnosis of retinitis pigmentosa (RP) underwent comprehensive ophthalmic examinations. Next-generation sequencing (NGS) was performed on samples from all patients. Detailed clinical characteristics of the patients with choroideremia identified in this study were assessed using multimodal imaging.

**Results:**

Seven patients with choroideremia were identified, and six novel variants in *CHM* (c.1960 T > C p.Ter654Gln, c.1257del p.Ile420*fs1, c.1103_1121delATGGCAACACTCCATTTTT p.Tyr368Cysfs35, c.1414-2A > T, and c.1213C > T p.Gln405Ter, c.117-1G > A) were revealed. All variants were deleterious mutations: two were frameshifts, two were nonsense mutations, two were splicing mutations, and one was a readthrough mutation. The clinical phenotypes of these patients were markedly heterogeneous, and they shared many common clinical features with RP, including night blindness, constriction of the visual field and gradually reduced visual acuity. However, patients with choroideremia showed pigment hypertrophy and clumping, and chorioretinal atrophy, and a majority of patients with choroideremia presented with retinal tubulations in the outer layer of the retina.

**Conclusions:**

We provide a detailed description of the genotypes and phenotypes of seven patients with choroideremia who were accurately diagnosed using NGS. These findings provide a better understanding of the genetics and phenotypes of choroideremia.

## Background

Choroideremia (OMIM 303100) is a rare, X-linked, recessive dystrophy that leads to the progressive degeneration of the retinal pigment epithelium (RPE), photoreceptors in the retina, and the choriocapillaris (CC) [1, 2]. The estimated prevalence is 1 in 50,000–100,000 individuals [3, 4]. Female carriers are affected with varying degrees of severity, but they are generally asymptomatic [5, 6]. Affected male patients suffer from nyctalopia, the progressive loss of the peripheral visual field and a reduction in the central visual field. Central visual acuity usually begins declining from the 30’s (4th decade) [7, 8]. Typically, patchy areas of chorioretinal degeneration occur in the mid periphery of the fundus, and these areas of degeneration proceed centripetally, eventually leading to the exposure of the underlying white sclera [2, 3, 9]. Therefore, choroideremia is often misdiagnosed as retinitis pigmentosa (RP) or other retinal dystrophies, such as Usher syndrome (OMIM 276900), gyrate atrophy (OMIM 258870) and Leber congenital amaurosis [10].

Choroideremia is caused by mutations in the *CHM* gene (OMIM, 300390), which encodes the geranylgeranyl transferase Rab escort protein-1 (REP-1). REP-1 deficiency affects intracellular vesicular trafficking, resulting in cellular dysfunction and premature cell death [11, 12]. *CHM* is located on chromosome Xq21.2, contains 15 exons, and encodes a 654-amino acid protein [13, 14]*. CHM* is the only gene known to be associated with choroideremia. To date, more than 280 mutations in the *CHM* gene have been reported to be associated with choroideremia, most of which are point mutations that directly introduce premature stop codons [12, 15]. However, the etiology and pathology of retinal degeneration caused by *CHM* mutations remains poorly characterized, and no phenotype-genotype correlations have been identified [2, 16, 17]. Gene therapy is a promising treatment option for choroideremia because it is caused by mutations in a single gene and the effective treatment window is relatively long; therefore, the impaired functions of the cells may be able to be reversed using gene-replacement therapy before the cells are irreversibly damaged [1, 18, 19]. Encouraging results from a recent phase 2 clinical trial showed that high-dose subfoveal gene therapy using an adeno-associated virus (AAV) expressing REP-1 (AAV2-REP-1) has the potential to maintain, and in some cases improve, the best-corrected visual acuity (BCVA) of patients with choroideremia [19].

The early and precise diagnosis of patients with choroideremia is vitally important for the selection of candidate patients who are suitable for gene therapy. Moreover, anatomical and functional evaluations of patients are necessary to determine the optimal intervention window and to predict and evaluate the potential therapeutic effects. In this study, seven patients with choroideremia were identified from 578 patients with a clinically suspected diagnosis of RP, and six novel variants in *CHM* were identified. The detailed clinical characteristics of the six patients carrying novel variants are described and analyzed here.

## Methods

### Subjects and ethics statement

This study was approved by the Ethics Committee of the Eye and ENT Hospital of Fudan University, Shanghai, China, and adhered to the tenets of the Declaration of Helsinki. All patients or their guardians provided written informed consent. Participants were screened at the Eye Genetic Disease Clinic of the Eye and ENT Hospital of Fudan University between January 2017 and December 2018.

### Clinical assessment

Study participants with suspected RP underwent comprehensively ophthalmic examinations after detailed family histories were obtained, including BCVA, slit-lamp biomicroscopic ophthalmoscopy exam, intraocular pressure (IOP, Goldmann tonometry), dilated fundoscopy, color fundus photography (Topcon TRC50LX; Topcon, Tokyo, Japan), swept-domain optical coherence tomography (SD-OCT, Spectralis HRAC OCT, Heidelberg, Engineering, Inc., Heidelberg, Germany), visual field (VF, Humphrey Visual Field Analyzer, Carl Zeiss Inc., Dublin, CA, USA), fundus autofluorescence (FAF, Spectralis HRA COCT; Heidelberg, Germany), full-field electroretinography (ERG, according to the standards of the International Society for Clinical Electrophysiology of Vision; available at www.iscev.org) and ultra-widefield scanning laser ophthalmoscopy (Optos 200Tx; Optos, England). VF was assessed by 30–2 Swedish Interactive Threshold Algorithm (SITA) Fast Programs to measure 30° temporally and nasally and test 76 points. The VF data were excluded if fixation loss and false-positive and false-negative response rates were greater than 20%. Average depression of visual sensitivity was estimated by mean deviation (MD). All patients accepted at least 5 VF examinations and were reviewed by 2 of us (G-Z X and Q C). Only patients with at least 5 reliable VF measurements were included.

### Genetic analysis and variant assessment

Exome sequencing was performed among all patients and available family members. Genomic DNA was extracted from whole peripheral blood using the FlexiGene DNA Kit (Qiagen, Venlo, the Netherlands) according to the manufacturer’s protocol. All participates underwent sequencing with a 762-gene (genes involved in common inherited eye diseases, Additional file 2: Table S1) panel (BGI, Shenzhen, China). It was designed to include the exon sequences of 762 genes ±30 bp, and reduce the impact of the reorganization. On average, the mean coverage depth was more than 300X and the coverage of target region was around 99.5% by using BGISEQ-500. We aligned sequence reads to the reference human genome (UCSC hg 38) with the Burrows-Wheeler aligner version 0.7.10 (BWA-MEM). The bioinformatics pipeline was applied as previously reported [20, 21]. Previous reported variants were determined using Clin Var (https://www.ncbi.nlm.nih.gov/clinvar/) and the Human Gene Mutation Database (HGMD, professional version 2019.1). Variants were classified as pathogenic, likely pathogenic, and variants of uncertain clinical significance according to the American College of Medical Genetics (ACMG) and genomics guidelines for the more recent cases^19, 20^. Sanger sequencing was performed to confirm variants within other members in the family.

## Results

### Cohort characteristics

**Fig. 1:**
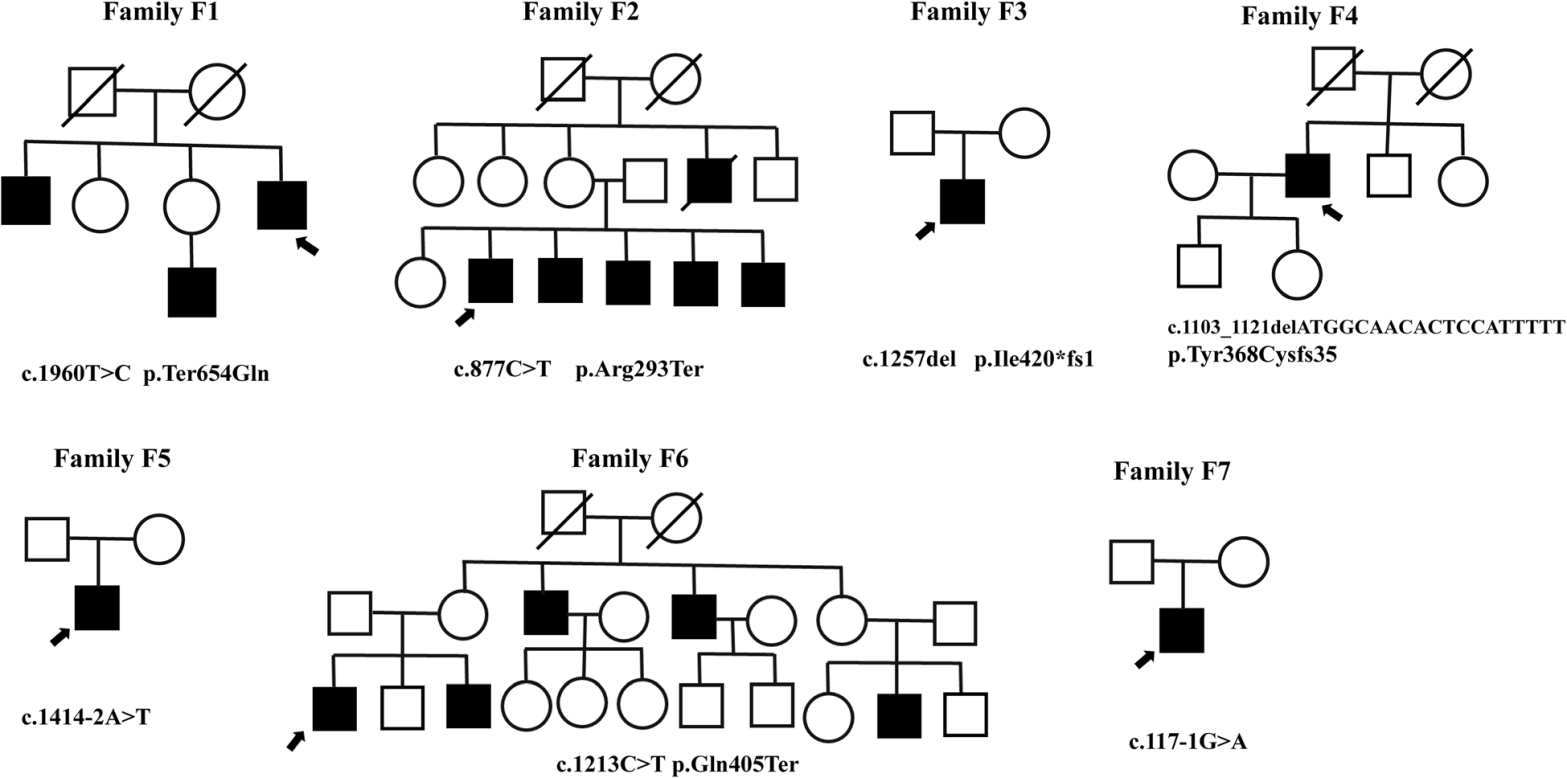
Pedigrees of the seven families in this study with *CHM* mutations. Filled symbols signify patients with choroideremia. Unfilled symbols represent unaffected family members. Arrows: probands; Squares: male individuals; Circles: female individuals. A slash indicates a deceased person

Five hundred seventy-eight patients with a clinically suspected diagnosis of RP and their available family members were recruited. We identified pathogenic or likely pathogenic causative variants within the *CHM* gene in seven patients from seven unrelated families. All patients were male. Accurate genetic diagnoses were determined after pedigree co-segregations and phenotypic confirmations were performed. Table 1 summarizes the clinical characteristics of the patients, and Fig. 1 presents the pedigrees of the seven families.

**Table 1:** Clinical characteristics of patients with choroideremia

Subjects	Age/onset	BCVA (R/L)	Refractive error (R/L)	MD (R/L, dB)	ERG
F1	67/65	0.5/0.5	−2.25/− 3.0	13.92/17.95	Profoundly attenuated rod ERGs, subnormal and delayed cone ERGs
F2	61/0	LP/LP	–	–	–
F3	28/18	0.6/0.5	−4.25/−5.0	28.1/27.2	Undetectable
F4	56/27	0.12/HM	−3.5/−3.0	33.6/33.9	Undetectable
F5	26/5	0.7/0.6	−8.0/−6.5	31.1/32.8	Undetectable
F6	34/0	0.6/0.8	−6.0/−5.0	32.4/32.13	Undetectable
F7	30/15	0.6/0.6	−2.5/−3.25	21.2/22.2	Undetectable

All patients were male. Abbreviations: *BCVA* best corrected visual acuity, *R* right, *L* left, -: unavailable, *LP* light perception, *HM* hand moving, *MD* mean deviation, *ERG* electroretinography.

### Genetic findings

Collectively, seven variants within the *CHM* gene (GenBank NM_000390.3) were identified (c.1960 T > C p.Ter654Gln, c.877C > T p.Arg293Ter, c.1257del p.Ile420*fs1, c.1103_1121delATGGCAACACTCCATTTTT p.Tyr368Cysfs35, c.1414-2A > T, c.1213C > T p.Gln405Ter, and c.117-1G > A), six of which were novel (Table 2). We did not identify a second pathogenic or likely pathogenic variant associated with inherited eye diseases in any patient. All seven variants are deleterious mutations: two are frameshift mutations, two are nonsense mutations, two are splicing mutations, and one is a readthrough mutation. No missense mutations were identified. Variants are distributed from exon 2 to exon 15.

**Table 2:** *CHM* variants identified in this cohort of patients

Subjects	Nucleotide Change	Amino Acid Change	Mutation Type	Exon/Intron	ACMG category	References
F1	c.1960 T > C	p.Ter654Gln	read through	EX15	LP	Novel
F2	c.877C > T	p.Arg293*	nonsense	EX7	P	[22, 23]
F3	c.1257del	p.Ile420*fs1	frameshift	EX10	LP	Novel
F4	c.1103_1121delATGGCAACACTCCATTTTT	p.Tyr368Cysfs35	frameshift	EX8	LP	Novel
F5	c.1414-2A > T	–	splicing	IN 11	P	Novel
F6	c.1213C > T	p.Gln405*	nonsense	EX8	P	Novel
F7	c.117-1G > A	–	splicing	EX2	LP	Novel

*P* pathogenic, *LP* Likely pathogenic

### Clinical findings

The clinical findings for all seven patients with choroideremia were highly suggestive of RP, and they were initially misdiagnosed with RP before the genetic analysis. However, further clinical and genetic analysis revealed choroideremia to be the correct diagnosis for these seven patients. Table 1 summarizes the clinical characteristics of the patients with choroideremia. Central vision was determined by light perception for patient F2; however, due to eccentric and insufficient fixation, some of the clinical findings for patient F2 were unable to be determined.

#### Disease onset and visual acuity

The exact age of onset is often difficult to determine. In this study, we used the time that the first symptom occurred as the onset age. The age of onset for patients F2, F5, and F6 was ≤5 years old. For patients F3, F4, and F7, onset occurred during adolescence or adulthood, and patient F1, a 67-year-old man, had only suffered from nyctalopia and vision loss for 2 years. The BCVA of patient F2 was light perception, while the BCVA of patient F4 was low vision (BCVA, 0.12/hand moving). The BCVAs of the other five patients were relatively good (≥ 0.5).

#### Ophthalmic and funduscopic findings

**Fig. 2:**
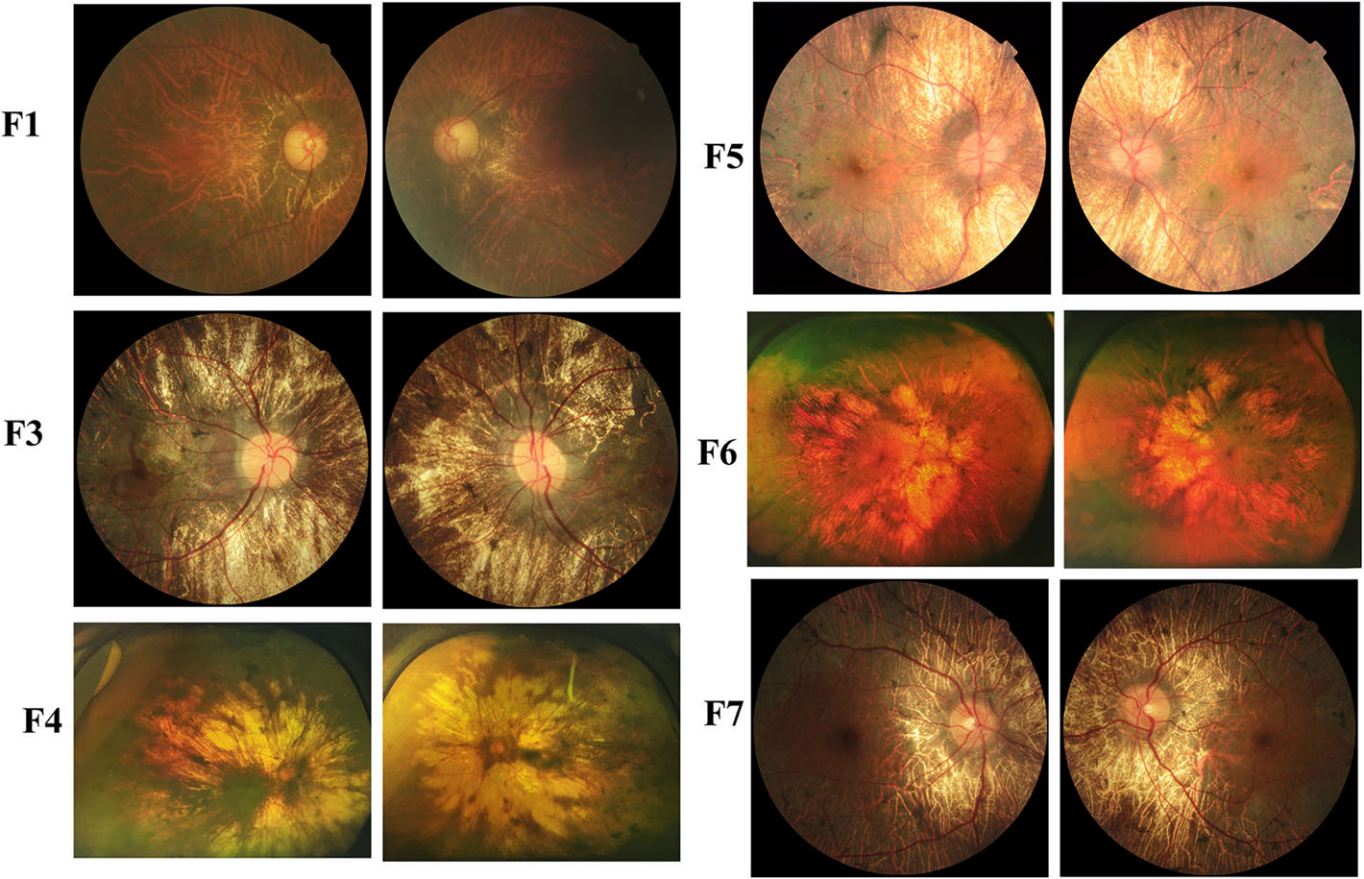
Fundus images of the six patients with choroideremia

Myopic refractive errors were observed in all six patients (Table 1). Patient F5 had severe myopia (− 8.0/− 6.5D), while the other five patients had low to moderate myopia. A fundus examinations of all patients revealed widespread loss of the RPE (Fig. 2). In patient F1, the RPE had degenerated extensively, with small islands of central retina persisting in the vascular arcade. Large choroidal vessels and vortex veins were visible, but bone spicules and a whitish fundus were not obvious. Patients F3, F4, F5, F6, and F7 showed the characteristic appearance of a whitish fundus, with large areas of profound atrophy in the choroid and retina. Large choroidal vessels and vortex veins were obvious, and multiple areas of pigment hypertrophy and clumping were observed. Retinal vascular attenuation was observed in patients F1, F4, F5, and F6.

#### Analysis of the retinal structure using SD-OCT

**Fig. 3:**
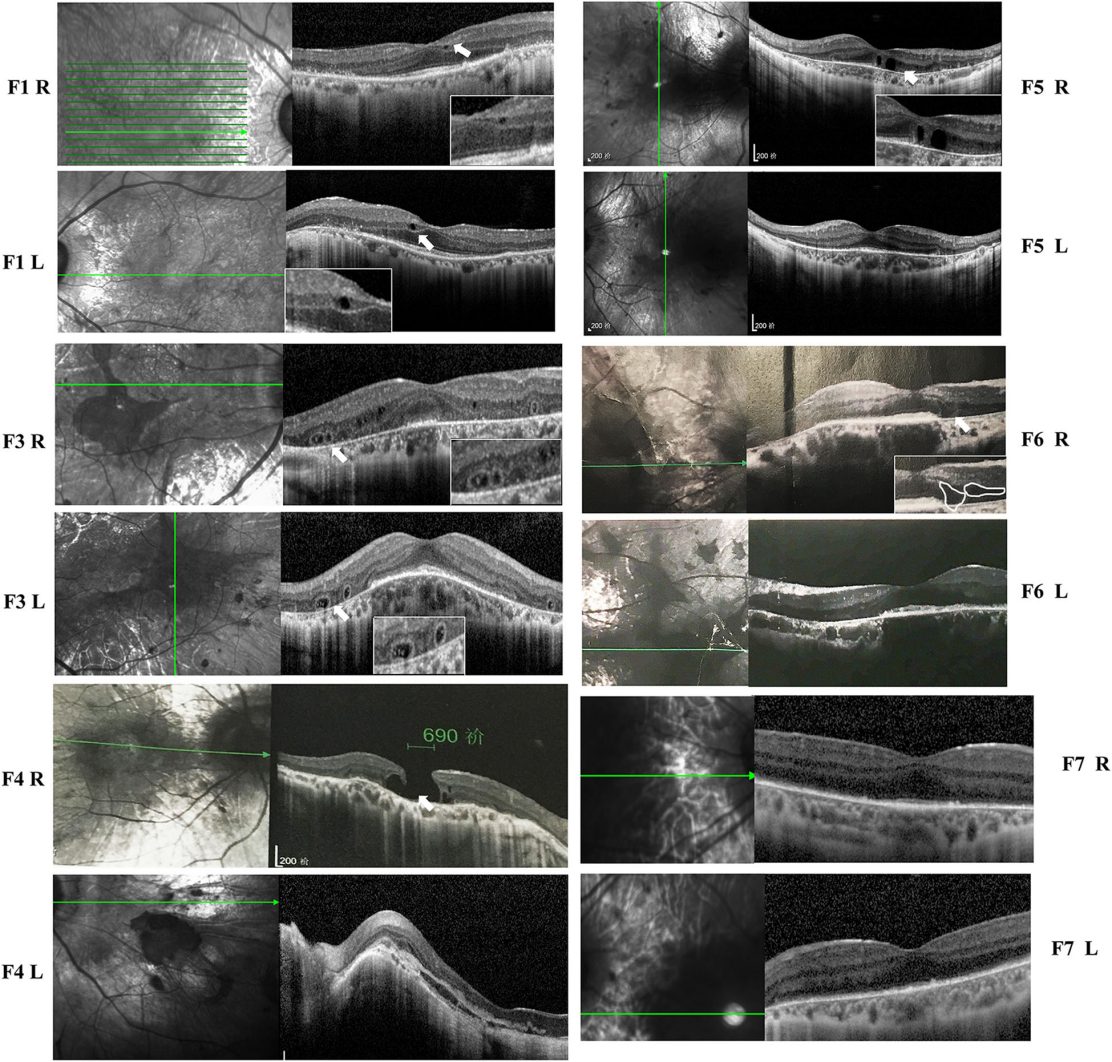
OCT images of the six patients with choroideremia. White arrows: tubulations or cystoid spaces. R, right; L, left

Different degrees of RPE degeneration, CC and choroid atrophy were observed in the SC-OCT images from all patients (Fig. 3). In patient F1, the RPE had completely degenerated up to the posterior pole, with the preservation of the ellipsoid zone, the external limiting membrane, and the outer nuclear layer. A small cystoid space was observed in the inner nuclear layer of the retina of both eyes. In patients F3, F4, F5, and F6, multiple outer retinal tubulations were observed in the outer nuclear layer of the peripheral macula. OCT images of patient F4 showed the complete absence of the RPE layer, the CC and the choroid. A full-thickness macular hole was observed in the right eye, and the normal structure and morphology of the macula were disordered in the left eye.

#### Analysis of retinal function by detecting the visual field and performing electroretinography

Humphrey static visual field analyses showed that only small central islands preserved in patients F3, F5, F6, and F7, while these areas of visual field had disappeared in patient F4 (Additional file 1: Figure S1). The specific value of MD was provided in Table 1 The visual field findings corresponded with extinguished electroretinography results in patients F3, F4, F5, F6, and F7, and were not different from the change in the visual field associated with RP. Uniquely, patient F1 presented a partial loss of the visual field, with only a loss of the mid-peripheral and superonasal visual field in both eyes. The retinal function of patient F1 was not completely lost, although the rod amplitude was profoundly attenuated and the cone amplitude was delayed.

## Discussion

With the development of gene sequencing technologies and the advent of gene therapy, genetic testing is becoming increasing attractive for patients with inherited retinal diseases [24, 25]. Genetic testing might be particularly vital for patients with choroideremia because choroideremia is often misdiagnosed as RP [10]. In addition, clinical trials using gene therapy to treat choroideremia have shown that gene therapy represents a promising treatment prospect [19, 26]. An accurate genetic diagnosis will provide support for a clinical diagnosis, modify future disease risks, and provide assistance for clinicians working in the field of retinal genetics. In addition, gene sequencing helps to identify suitable candidates for future choroideremia gene therapy trials. In the present study, seven patients who were previously diagnosed with RP received corrective and accurate diagnoses of choroideremia based on NGS.

Six of the seven variants identified in this study were novel. All of the variants were deleterious mutations, and no missense mutations were identified. This result was similar to the findings reported in other countries, such as America [27], Europe [22], and Canada [28]. This finding implied that most missense mutations in CHM exert little or no effect on the protein structure and function. However, deleterious mutations exert substantial effects on the structure of the encoded protein, potentially leading to a loss of GGTase function and resulting in the insufficient transfer of geranylgeranylpyrophosphate groups onto Rab proteins, inducing CHM [14, 29]. These results not only expand the mutational spectrum of *CHM* but also suggest an important feature of *CHM* mutations in this cohort with relevant guiding value for genetic counseling. These results also are important in guiding the selection of sequencing methods used for patients with choroideremia. Various sequencing methods have been used to identify *CHM* mutations, with Sanger sequencing and NGS representing the most widely used methods [30–32]. However, mutations in the *CHM* gene are often not identified. One possible reason is that copy number variations and structural variations are important pathogenic mutations observed in patients with choroideremia and should be considered during routine genetic sequencing, particularly when small mutation analyses produce negative results. Moreover, no hotspots or hot regions were detected among the *CHM* mutations, and these results have important value for guiding genetic counseling.

Clinically, choroideremia is often misdiagnosed as RP. Approximately 6% of individuals diagnosed with RP might instead have choroideremia [33, 34], while approximately one-quarter of patients with a clinical diagnosis of choroideremia may actually have other diseases, including RP [34]. In the present study, we analyzed and reported the clinical characteristics of patients with choroideremia, and we summarized the detailed differences between choroideremia and RP based on a multimodal imaging analysis of the phenotypes among the six patients. First, unlike RP, patients with choroideremia do not show ‘bone spicules’ pigmentation; instead, they show pigment hypertrophy and clumping. However, the specific types of RP, such as pigment-free or advanced stage RP, can be difficult to identify in patients. Second, the characteristic appearance of patients with choroideremia is a whitish fundus with large areas of profound atrophy observed in the choroid and the retina, and large choroidal vessels and vortex veins are obvious. However, in the early stages of disease, the differentiation between RP and choroideremia may be difficult, as the choroid degeneration may only become apparent during later stages [35]. Third, most of the patients with choroideremia examined in this study presented with better visual acuity than patients with RP; however, this finding cannot be generalized because the vision prognoses among patients with different types of RP vary substantially [36, 37]. Fourth, the majority of patients with choroideremia present with retinal tubulations in the outer layer of the retina. Although the underlying pathogenesis remains unclear, researchers have postulated that these tubulations may represent the tubular rearrangement of degenerating photoreceptors [38–40]. These tubulations are present around areas of the surviving retina and are thought to represent areas that retain some visual function [10, 41]. Therefore, due to the high level of phenotypic heterogeneity among patients with choroideremia, the clinical analysis described above should only serve as a reference. The “gold standard” for diagnosing choroideremia must be based on a genetic diagnosis. Moreover, *CHM* should be included as a candidate gene during gene sequencing of patients suspected of having RP.

The limitations of this study include the small number of patients, and more patients with diverse pathological phenotypes and longer follow-up periods are necessary.

## Conclusions

In conclusion, we provided a detailed description of the genotypes and phenotypes of seven patients with choroideremia who were accurately diagnosed using NGS. Six novel mutations were identified, which not only expanded the mutational spectrum of *CHM* gene, but also improve our understanding of choroideremia.

## Supplementary information

**Additional file 1: Figure S1.** Humphrey static visual field analysis of the six patients with choroideremia.

**Additional file 2: Table S1.** Details of the 762 genes in the panel.

## Data Availability

All data relevant to the study are included in the article or uploaded as supplementary information.

## Abbreviations

RP: Retinitis pigmentosa
NGS: Next-generation sequencing
RPE: Retinal pigment epithelium
CC: Choriocapillaris
REP-1: Rab escort protein-1
BCVA: Best-corrected visual acuity
IOP: Intraocular pressure
SD-OCT: Swept-domain optical coherence tomography
HGMD: Human Gene Mutation Database
ACMG: American College of Medical Genetics
