# Supplementary figures and images for: Next-generation sequencing-based clinical diagnosis of choroideremia and comprehensive mutational and clinical analyses
Source: BMC Ophthalmol. 2020 Jun 1;20:212. doi: 10.1186/s12886-020-01478-x (PMC7268499; doi:10.1186/s12886-020-01478-x)

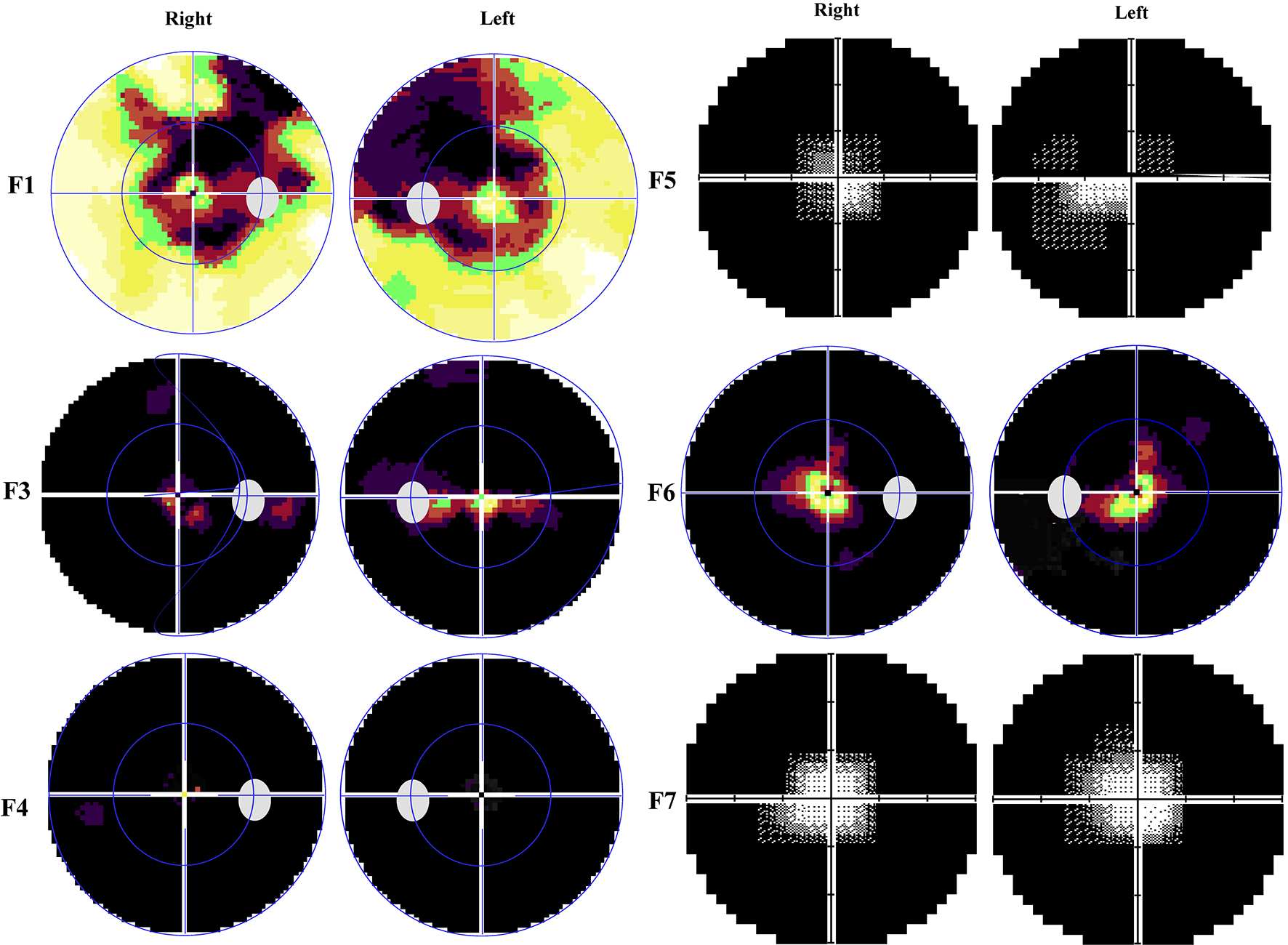

Supplement: Supplementary file 1 — Additional file 1: Figure S1. Humphrey static visual field analysis of the six patients with choroideremia. [file 12886_2020_1478_MOESM1_ESM.tif]
